# The Content and Size of Hyaluronan in Biological Fluids and Tissues

**DOI:** 10.3389/fimmu.2015.00261

**Published:** 2015-06-02

**Authors:** Mary K. Cowman, Hong-Gee Lee, Kathryn L. Schwertfeger, James B. McCarthy, Eva A. Turley

**Affiliations:** ^1^Department of Chemical and Biomolecular Engineering, Biomatrix Research Center, New York University Polytechnic School of Engineering, New York, NY, USA; ^2^Department of Laboratory Medicine and Pathology, Masonic Comprehensive Cancer Center, University of Minnesota, Minneapolis, MN, USA; ^3^Department of Oncology, London Health Sciences Center, Schulich School of Medicine, Western University, London, ON, Canada; ^4^Department of Biochemistry, London Health Sciences Center, Schulich School of Medicine, Western University, London, ON, Canada; ^5^Department of Surgery, London Health Sciences Center, Schulich School of Medicine, Western University, London, ON, Canada

**Keywords:** hyaluronan, quantification, assay, molecular mass, molecular weight

## Abstract

Hyaluronan is a simple repeating disaccharide polymer, synthesized at the cell surface by integral membrane synthases. The repeating sequence is perfectly homogeneous, and is the same in all vertebrate tissues and fluids. The polymer molecular mass is more variable. Most commonly, hyaluronan is synthesized as a high-molecular mass polymer, with an average molecular mass of approximately 1000–8000 kDa. There are a number of studies showing increased hyaluronan content, but reduced average molecular mass with a broader range of sizes present, in tissues or fluids when inflammatory or tissue-remodeling processes occur. In parallel studies, exogenous hyaluronan fragments of low-molecular mass (generally, <200 kDa) have been shown to affect cell behavior through binding to receptor proteins such as CD44 and RHAMM (gene name HMMR), and to signal either directly or indirectly through toll-like receptors. These data suggest that receptor sensitivity to hyaluronan size provides a biosensor of the state of the microenvironment surrounding the cell. Sensitive methods for isolation and characterization of hyaluronan and its fragments have been developed and continue to improve. This review provides an overview of the methods and our current state of knowledge of hyaluronan content and size distribution in biological fluids and tissues.

## Introduction

Hyaluronan (hyaluronic acid, HA) is found in vertebrate tissues, as a key component of the extracellular matrix. It has a simple covalent structure consisting of alternating β-d-glucuronate and *N*-acetyl-β-d-glucosamine sugars. The linear anionic polymer has a semi-flexible structure, causing it to adopt an expanded wormlike random coil. The domain of a coiled chain can be conceptually described as a sphere [mostly filled with (unbound) solvent], with a dynamically changing chain configuration. The apparent volume occupied by a single isolated molecule depends strongly on the chain length, and thus the molecular mass, *M*. The volume increases approximately as *M* raised to the 1.8 power, as dictated by polymer chain statistics (1). Where HA chains are crowded together, their domains are forced to interpenetrate, and this leads to severe non-ideality in behavior. The non-ideality determines such properties as the large colloid osmotic pressure, viscoelasticity, and effect on partition of other macromolecules (excluded volume) in the biomatrix. Since the molecular mass of HA in normal biological fluids and tissues is normally very high (ca. 1000–8000 kDa), the non-ideality effects dominate the physicochemical properties of HA in the extracellular matrix (2–4).

In addition to the physicochemical effects due to mutual macromolecular crowding, HA has important binding interactions. At the cell surface, HA provides a protective coat that is tethered to receptors embedded in the cell membrane (5–7). Beyond the cell surface, HA organizes proteoglycans (e.g., versican, aggrecan) and other binding proteins via specific non-covalent interactions, creating a further highly hydrated and charged domain (8–10). In inflammation and other specific tissue-remodeling processes, covalent transfer of the heavy chain domains of IαI to HA can be catalyzed by TSG-6 protein (11–14). The HA-protein assemblies, whether covalently or non-covalently mediated, are integral to maintenance of an expanded pericellular matrix.

Normally, HA has a high turnover rate (15, 16). Thus, the extracellular environment is constantly renewed. The need for renewal may reside in the protective role HA fulfills. Facile degradation of HA by reactive oxygen and nitrogen species (ROS/RNS) (17, 18) during active inflammation can weaken the protective HA coat that usually protects the cell. The HA acts as a scavenger of damaging free radicals and other chemical agents. If the rate of HA synthesis keeps pace with the rate of degradation and turnover, the homeostatic environment is maintained (19).

When the rate of HA degradation is not adequately compensated by its synthesis, fragments of the polymer might be present at significant levels and consequently cells are poorly protected. Changes in the physicochemical control of the pericellular environment take place. HA fragments compete in binding interactions with proteins, altering the integrity of the biomatrix. Fragments can displace high *M* HA in interactions with cell surface receptors, resulting in changes in receptor clustering and altered signaling (20). Fragments can also signal through alternate receptors (21–23). In these ways, HA may be regarded as a biosensor of damaging processes in the cellular microenvironment. Altering the balance of high and low *M* HA is a stimulus that sets in motion multiple cellular response mechanisms. These can be purely defensive, such as HA fragment-induced expression of β-defensins to combat microbial infection in the gut (24, 25). But sustained responses can also lead to chronic inflammation via aberrant signaling through receptors and consequently increased expression of inflammation mediators (26).

Tissue-remodeling processes, including wound healing and tumor progression, are associated with changes in HA content and size (27–31). HA synthesis is usually increased during remodeling, but increased expression of hyaluronidases may also occur, and together with macrophage-generated ROS/RNS, degrade HA. The balance of high and low *M* HA may differ from the homeostatic case, thus altering both the physicochemical and signaling effects of HA. To understand HA biology, we are faced with multiple questions: (1) What is the content of HA present, (2) What is the molecular mass distribution of the HA, and (3) Can we control pathological processes by altering the content, size, and binding interactions of HA?

## Isolation of HA

There are a number of methods appropriate for assaying the content and size of HA in biological fluids and tissues. Depending on the method, it may be necessary to purify HA to remove/digest bound proteins and sulfated glycosaminoglycans prior to assay.

The isolation of HA follows protocols (25, 30, 32–35) that are quite similar to those historically employed in the purification of DNA. The requirement for specific steps depends on the nature of the sample: fluid tissue vs. conditioned medium from cell culture vs. solid tissue. For solid tissues, the HA is extracted into soluble form, and liberated from proteins. Protein removal can be accomplished by digestion with a protease, or by denaturing the protein by gentle mixing with chloroform. Lipids are removed with acetone or other organic solvent mixtures. Removal of low-molecular mass contaminants may require dialysis, or precipitation of the HA with ethanol or isopropyl alcohol. DNA and RNA can be enzymatically digested. There are many variations on these steps. A sample protocol for extraction of HA from solid tissue might include the following steps: digestion with a protease such as proteinase K, boiling to denature enzyme, centrifugation, extraction with chloroform, centrifugation, dialysis, precipitation with ethanol, centrifugation, re-dissolution, digestion with Benzonase (or DNase plus RNase), boiling to denature enzyme, and repeat of steps starting with chloroform extraction. Abbreviated protocols can be used for fluid samples, or where the HA needs to be liberated but not purified because specific assay will be employed.

The above purification will not remove other glycosaminoglycans. Sulfated glycosaminoglycans can be removed by anion exchange chromatography. Unsulfated or undersulfated chondroitin, which is rare in normal tissues but may be significant in remodeling tissues, is not removed by this process. At this point in the procedure, specific isolation of HA can be accomplished by affinity methods, such as use of a biotinylated HA-specific binding protein and streptavidin-coated magnetic beads, or other similar medium such as gel beads (36, 37).

It is worth noting that most isolation methods in current use have not been validated with respect to quantitative yield of HA, or preferential extraction/isolation of specific HA sizes. In particular, losses of very low *M* HA may be significant in some procedures. It is also possible to degrade HA during isolation. Endogenous enzymes may cause some of this degradation. However, most degradation is the result of ROS generation catalyzed by contaminating iron (II) or copper (I), and molecular species that regenerate the active metal ion oxidation states. Thus, use of papain activated with cysteine can lead to HA degradation (38). The presence of EDTA can also enhance the ability of contaminating iron to catalyze formation of hydroxyl radicals. Iron contamination is better inactivated by chelation with deferoxamine (34). Also, of note, EDTA and phosphates can be co-precipitated with HA using ethanol.

Testing for degradation of HA during isolation can be easily accomplished by “spiking” the initial fluid or tissue with a pure HA sample of known *M* and low polydispersity in *M*, and then testing its size in the final isolate. Spiking samples with known amounts of HA can also be used to detect losses during isolation, including losses due to non-specific interactions with surfaces or other macromolecules that HA may not normally contact.

## Methods to Analyze Content of HA

The most simple and historical assay for HA is measurement of uronic acid content. The assay involves hydrolysis in concentrated sulfuric acid, so that protein content is not a problem. Other glycosaminoglycans that contain uronic acid will contribute to the result, and should be separated or removed from HA if possible. The uronic acid assay has been widely employed, especially in analysis of fluids (synovial fluid, vitreous) with high HA and low sulfated glycosaminoglycan content.

Hyaluronic acid content can be also determined by analysis of the oligosaccharide products of enzymatic digestion. Quantification is accomplished by methods such as HPLC, capillary electrophoresis (CE), mass spectrometry, or fluorophore-assisted carbohydrate electrophoresis. These methods have primarily been employed to determine relative amounts of different glycosaminoglycans in a sample, rather than absolute quantities.

The most sensitive, specific, and accurate methods for determination of HA content are based on enzyme-linked sorbent assays (ELSA, ELISA-like assays) (39–47). The specific detection of HA is an important step of these methods, because purification of low *M* HA is difficult, and contaminants interfere with non-specific detection modes. The specificity is based on the use of molecular species such as proteins or proteoglycans that recognize and bind HA but no other biological molecules. For example, the aggrecan proteoglycan binds HA specifically (8, 9). The intact proteoglycan may be used, or a terminal fragment called globular domain 1 – interglobular domain – globular domain 2 (G1–IGD–G2), often referred to as HA-binding protein (HABP) or HA-binding region (HABR). The link protein, also called CRTL1 or HAPLN1, is similar to the G1 domain of aggrecan, and is another suitable protein for specific detection of HA. Isolated HABP, usually a mixture of the aggrecan HABR and the link protein, may also be used. Similarly, versican proteoglycan G1 domain is useful. Hyaluronectin, a HA-specific binding protein isolated from brain, may be used. Recently, a recombinant fusion protein of human TSG-6 and the Fc domain of human IgG, and a second variant of the fusion protein in which the heparin-binding region of TSG-6 was mutated to become inactive, were found to be suitable for development of a specific HA assay (47). Other HA-specific binding molecules could be used.

There are two types of ELSA. The first type, sandwich assays, are sensitive and reproducible, but fail to adequately quantify low *M* HA (39–42). This is because the plate surface, coated with an HABP, strongly binds HA, but does not allow further probing of short HA chains by the detector protein (43). Longer HA chains have accessibility as a result of looped sections above the surface. (The same problem occurs in HA blotted to positively charged nylon membranes after electrophoresis, from which short HA chains cannot be detected.) The second type of ELSA is competitive assays. In these, HA is usually immobilized on a surface such as the wells of a plastic 96-well plate. Alternative surfaces are suitably modified magnetic beads. Soluble HA samples, either standards or unknowns, are mixed with the specific binding agent, usually a protein or proteoglycan. The soluble HA competes with immobilized surface HA for the specific binding agent, so that the resulting surface-bound amount of the binding agent is a measure of the amount of soluble HA in the sample being analyzed (41, 44, 45). There are multiple possible detection schemes to quantify the bound agent. For example, if aggrecan proteoglycan is the specific binding agent, it can be quantified with an antibody to the keratan sulfate chains of aggrecan, and a suitably labeled second antibody. When the specific binding agent is a labeled (e.g., biotinylated) HABP, it can be quantified by binding of the label to a specific agent such as streptavidin, which is, in turn, conjugated to an enzyme or other detectable species. Radiolabeled HABP may also be used but is less desirable on the basis of safety and disposal. Because the recognition step in a competitive binding assay occurs in solution, HA chains as short as approximately decasaccharides can be accurately detected, depending on the labeled binding protein used. The results of sandwich and competitive assays have been shown to be in good agreement for high *M* HA (46).

## HA Content in Biological Fluids and Tissues

The content of HA in many normal biological fluids has been determined. Here, we cite a few relevant results. HA is a major component of articular joint synovial fluid, where it provides the viscoelasticity and lubrication necessary for protection of cartilage surfaces. Its concentration in the human knee joint is approximately 2–3 mg/ml, being slightly higher in younger adults than in older adults (48–50). HA is also a major component of the vitreous body of the eye, but at a lower concentration of approximately 200 μg/ml, in the phakic human eye vitreous (51). The concentration in the aqueous humor is lower still, being only about 1 μg/ml (52). Human lymph fluid contains HA at a concentration of about 0.1–18 μg/ml (36). In the blood serum of healthy human adults, the concentration of HA is lower still, being usually between 10 and 100 ng/ml, mostly 20–40 ng/ml, and averaging about 30 ng/ml (36, 40, 44). Normal human urine also contains a low level of HA, around 100–300 ng/ml (44), and human milk similarly contains HA at about 200–800 ng/ml (25).

The HA content of solid tissues varies widely. Bovine nasal cartilage contains approximately 1200 μg HA/g wet tissue weight (44). The HA content of human articular cartilage is similar, being about 500–2500 μg/g (53). Human skin contains approximately 400–500 μg HA/g tissue, mostly in the dermis (54). Fetal skin and young skin have higher HA contents than older skin. Other organs have much less HA. Laurent and Tengblad (44) reported HA contents of approximately 1–100 μg HA/g wet tissue weight for most organs. Rabbit kidney had 103 μg/g, brain had 65 μg/g, muscle had 27 μg/g, liver had 1.5 μg/g, and cornea had 1.3 μg/g. Armstrong and Bell (34) also reported rabbit tissue HA contents of 500 μg/g for skin, 200 μg/g for large intestine and heart, 130 μg/g for small intestine, and 80–90 μg/g for lung and muscle tissues.

Measurement of HA content is of continuing high interest, because there are multiple studies correlating changes in HA content with tissue remodeling and pathological processes. While the normal HA concentration in human serum is usually <40 ng/ml, it is elevated (>46.5 ng/ml) in hepatic cirrhosis (55), in rheumatoid arthritis (56, 57) (highly variable; reports up to nearly 200 μg/ml, but more generally between 0.07 and 6.4 μg/ml), in ankylosing spondylitis (57) (7–13 μg/ml), and in osteoarthritis (57, 58) (0.04–2.3 μg/ml). The elevated HA concentration in serum of patients with hepatic cirrhosis is utilized as one component of a diagnostic assay. A small but significant elevation (frequently, about twofold) of HA in serum is found in multiple types of untreated cancer (59–61). Radical surgery to remove the tumor causes the HA concentration in serum to return to the normal range. Most interestingly, it was found that the low-molecular mass component of serum HA can be used to differentiate metastatic from non-metastatic breast cancer (62), which may form the basis of a new diagnostic test.

In solid tissues, many but not all cancers progress in a tumor microenvironment of increased HA content (28). Further, some non-aggressive cancer types such as non-malignant fibroadenoma produce elevated HA (63, 64). The presence of HA may therefore not be sufficient by itself to promote tumorigenesis. However, high levels of HA accumulate in lung, colorectal, prostate, bladder, and breast carcinomas and in these cancers are linked to tumor aggression (28). For example, the HA content of human lung tissue increases 4- to 200-fold in lung carcinoma (65), 100-fold in grade 3 ovarian cancer (66) and 7-fold in prostate cancer (67). Increased tumor HA accumulation is also linked to tumor aggression. The HA content of malignant ovarian epithelial tumor correlates with tumor grade and with metastasis. Elevated HA accumulation within the stroma or tumor parenchyma of breast cancer is associated with unfavorable prognosis of the patient. Recent studies have further linked high stromal HA staining to HER2 positive tumors and poor outcome parameters including time to relapse, large tumor size, lymph node positivity, hormone receptor negativity, high body mass, and shortened overall survival (68). Elevated HA in the tumor microenvironment is linked to inflammation (69). Thus, high amounts of both tumor-associated macrophages and HA are concurrent in breast carcinoma. High macrophage numbers correlate with high tumor HA, HAS expression and poor outcome, suggesting that HA facilitates a macrophage tumor supporting function in breast cancer. The link between inflammation and cancer has led to recent interest in HA as a contributor to a pro-tumorigenic inflammatory environment, as detailed in a companion article in this issue (70).

As for cancer, wound healing and fibrosis are associated with inflammation and increased HA content (71). An approximately twofold increase was observed in HA content of rat skin during healing of excisional wounds (30). Similarly, scleroderma patients with early stage disease have an approximately twofold increase in serum HA (72). Many other pathological states characterized by inflammation similarly have elevated HA, as estimated by immunohistochemical analyses (73, 74).

## Methods to Analyze HA Molecular Mass Distribution

It has long been appreciated that degradation of HA negatively affects its biomechanical properties. For example, degradation of HA in articular joint synovial fluid can reduce the viscosity and elasticity of the synovial fluid, and has also been shown to reduce its lubricating ability (49, 75). The widespread and successful uses of solutions of high molecular mass HA as a viscosurgical tool in ophthalmic surgery, and as an analgesic treatment for osteoarthritis, are based on this understanding. More recently, the discovery that exogenous HA fragments can alter cellular behavior by signaling through multiple receptor proteins, and that the existence of such fragments *in vivo* is likely, based on increased hyaluronidase levels and reactive oxygen and nitrogen species in tissue remodeling and pathological processes, has led to increased interest in measuring the size distribution of HA in biological fluids and tissues.

Many current methods for determination of the *M* distribution of HA from tissues and biological fluids have been optimized for highly purified HA. A commonly employed method used commercially is size exclusion chromatography with multiangle laser light scattering (SEC-MALLS) (76, 77). However, detection of very low *M* HA by light scattering is inherently insensitive, and the SEC-MALLS method requires a highly purified HA sample. CE (78) is similarly limited to pure HA samples. MALDI-TOF mass spectrometry (79, 80) has high sensitivity, but requires a pure sample and HA with *M* larger than about 10 kDa becomes difficult to analyze. A new method that has extremely high sensitivity and works best for low *M* HA is gas-phase electrophoretic mobility molecular analysis (GEMMA), but it still requires pure HA (81).

The most widely used methods, to date, for size distribution analysis of imperfectly pure HA isolated from biological samples are size exclusion chromatography with enzyme-linked sorbent assay (SEC-ELSA) (36, 56, 82), and agarose or polyacrylamide gel electrophoresis (83–86) with staining or with blotting and specific detection. Both methods are capable of detecting a wide range of HA sizes. Gel electrophoresis with staining can analyze samples on the microgram scale, and can tolerate some impurities in the sample, but non-specific staining by those impurities can interfere with size distribution analysis of the HA. Blotting of gels to positively charged nylon and detection of HA using a labeled specific binding protein works only for HA with *M* >100 kDa, as a result of strong surface binding (43). To address the issues of limited sample amount, purification difficulty, and the importance of analyzing both high and low *M* HA simultaneously, we recently developed a method using size-dependent fractionation of HA by anion exchange on a spin column, and quantification of HA in the fractions using a competitive ELSA assay (IEX-ELSA) (37). All of these methods require calibration with purified HA samples of known size.

## HA Size in Biological Fluids and Tissues

The average *M* and distribution of *M* for HA present in biological sources have been studied primarily for fluid tissues such as synovial fluid, vitreous, serum, lymph, and milk. Until recently, the emphasis has been on documenting reduction of the average *M*, which strongly affects the biomechanical properties of HA solutions (48, 87–89). This has been done using physicochemical methods such as viscometry, light scattering, and sedimentation–diffusion. Interest in the distribution of sizes present, and the possibility that specific sizes have unique biological effects, has led to an increasing number of studies by chromatographic and electrophoretic separation methods.

**Figure 1:**
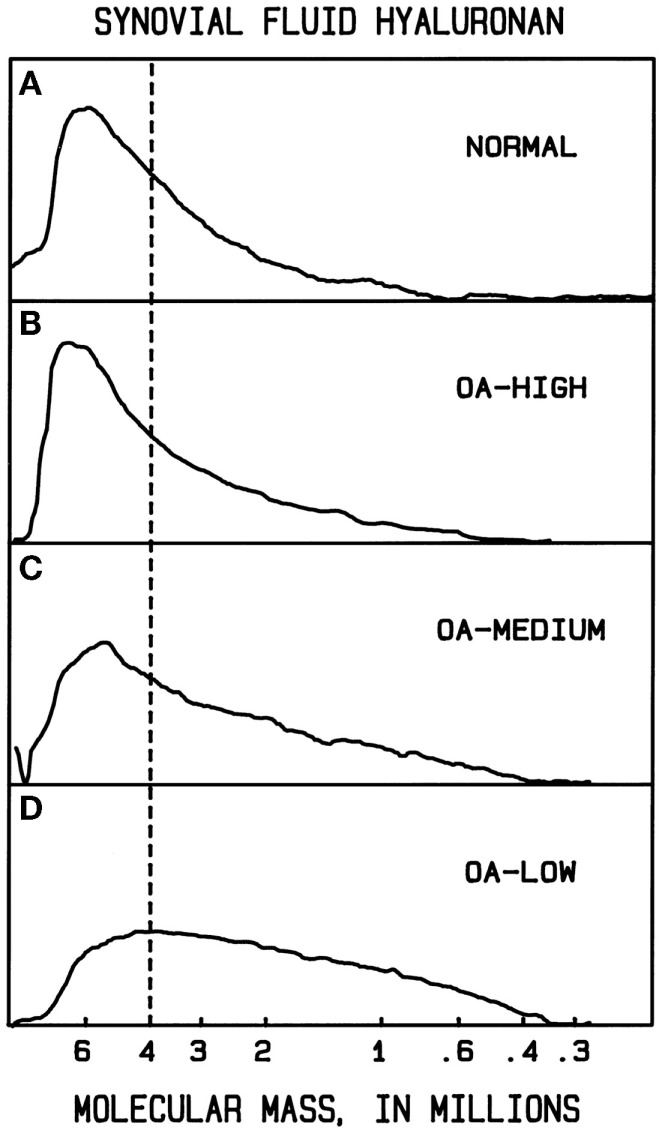
**Example molecular mass distributions of human synovial fluid (SF) HA determined by agarose gel electrophoresis**. From top to bottom: **(A)** normal human SF obtained from young healthy volunteers. **(B–D)** Representative osteoarthritis (OA) patient SF. The dashed vertical line corresponds to the migration position of 4000 kDa HA. The fraction of HA having slower electrophoretic migration, and thus higher *M* than 4000 kDa, is a measure of the high *M* HA content. In normal human SF, the portion of HA with *M* > 4000 kDa averaged 61%. OA patients varied in the extent of HA degradation. The OA-HIGH profile, similar to that seen in normal SF and representative of OA samples with more than 60% of HA having *M* > 4000 kDa, was found for 26% of patients. The OA-MEDIUM profile, representative of OA samples with approximately 41–60% of HA having *M* > 4000 kDa, was found for 41% of patients. The OA-LOW profile, representative of OA samples with <40% of HA having *M* > 4000 kDa, was found for 33% of patients. From Lee (90).

In normal human synovial fluid, most of the HA is very high in molecular mass. Gel filtration chromatography with HA-specific detection (50) and agarose gel electrophoresis with staining (84, 90) show the average *M* to be approximately 6000–7000 kDa, with little if any HA <1000 kDa. In rheumatoid arthritis and in osteoarthritis, HA can be partially degraded, resulting in a broad distribution of sizes, extending perhaps down to a few hundred kilodaltons (90, 91) (Figure 1). Normal rabbit vitreous HA has mostly high *M* (2000–3000 kDa), but bovine vitreous HA has mostly moderate *M* (500–800 kDa) (82). Owl monkey vitreous has very high *M* HA (84).

For fluids containing HA at very low concentrations, determination of the *M* distribution is correspondingly difficult. Despite this, evidence for the occurrence of HA below 100 kDa (<250 disaccharides) in *M* is accumulating. Human milk contains mainly HA with an average *M* of about 440 kDa, and also has been definitively shown to have approximately 5% of HA with *M* < 100 kDa (37). The low *M* HA is proposed to participate in stimulating the expression of human β-defensin 2 in the infant intestinal epithelium (24). Human amniotic fluid contains HA with an average *M* of about 330 kDa at 16 weeks gestation, but the *M* distribution changes to a mixture of high and very low *M* HA by 40 weeks gestation (92). HA in lymph fluid is variable in size, and can occur as a mixture of high and lower *M* components (36), or as a broad distribution of moderate *M*, ca. 800 kDa average (93). HA in normal blood serum is mainly relatively low *M* (ca. 100–300 kDa) (36, 56). It is also low in *M* in saliva and urine (94, 95).

Tumors have been proposed to shed very low *M* HA into associated body fluids. The quantity of such very low (but undetermined size) *M* HA in patient serum, obtained by centrifugal filtration, has been reported to be associated with metastatic breast cancer (62). It has also been reported in saliva of patients with head and neck tumors (95), and in the urine of patients with bladder cancers (94). The precise size of all such HA has not yet been determined, but should be accessible using recent improvements in methods. Rarely, high *M* HA is found in serum, as, for example, associated with Wilm’s tumor (96).

For solid tissues, the pattern is a bit simpler. Normal healthy tissues are almost always associated with high *M* HA. HA with average *M* > 2000 kDa is found in young human cartilage (53). Larger HA averaging closer to 4000–6000 kDa is found in human skin (54), in rabbit skin (34), and in rat skin (30, 97). High *M* HA is found in rooster combs (32). High *M* HA is also found in skeletal muscle, lung, heart, ileum, and colon of the rabbit (34). Little if any low *M* HA is found in these healthy tissues.

Remodeling tissues and tumors show evidence of some lower *M* HA. Reduction in HA *M* occurs in older human cartilage (53). Low *M* HA also occurs in healing rat skin wounds (30), in human skin following irradiation with UVB (74), and in mouse cervix undergoing postpartum remodeling (98). It is found in rat kidney after ischemia–reperfusion injury (99). Human prostate tumor HA has also been reported to contain some low *M* HA of indeterminate size (67). Many of the above-described studies of reduced *M* HA should be regarded as indicative but not conclusive proof of the presence of specific low *M* HA species. Recent improvements in techniques for analysis of very low quantities of polydisperse HA will allow this uncertainty to be addressed. Future studies should also include spiking samples with multiple monodisperse HA species to show that the isolation methods cause no degradation, or preferential isolation of high or low *M* HA.

It is interesting to consider that all efforts to determine the content and size of HA in biological tissues and fluids have made the tacit assumption that the HA has a constant chemical structure, except for variation in size. Since degradation by ROS/RNS can cause chemical changes including ring opening reactions, it is possible that HA assays and size analyses may be influenced by such changes, if present at significant levels. Further examination of this possibility is warranted.

## Conclusion

It is now well established that HA synthesis is significantly increased in remodeling tissues and tumors. The concomitant presence of hyaluronidases and ROS/RNS makes it likely that fragments of HA can be created by degradation of high *M* polymers. The balance of synthetic and degradative activities, coupled with turnover through outflow or internalization, will determine the steady state *M* distribution of the tissue HA. HA shed into lymph or blood from a tumor may represent only the lowest *M* fraction of that present. Whether HA fragments of particular sizes exist in sufficient amounts within a tissue or tumor environment to trigger specific cellular responses is not yet clear. The fact that exogenous HA fragments can elicit such effects is suggestive but not yet a proof of their role *in vivo*. There is good reason to expect clarity on these issues in the near future.

## Author Contributions

MC, HL, KS, JM, and ET contributed to the drafting and revising of this manuscript. All authors approved this manuscript.

## Conflict of Interest Statement

The authors declare that the research was conducted in the absence of any commercial or financial relationships that could be construed as a potential conflict of interest.
